# Measurement of Glomerular Filtration Rate using Quantitative SPECT/CT and Deep-learning-based Kidney Segmentation

**DOI:** 10.1038/s41598-019-40710-7

**Published:** 2019-03-12

**Authors:** Junyoung Park, Sungwoo Bae, Seongho Seo, Sohyun Park, Ji-In Bang, Jeong Hee Han, Won Woo Lee, Jae Sung Lee

**Affiliations:** 1 Department of Biomedical Sciences, Seoul National University College of Medicine, Seoul, Korea; 2 Department of Nuclear Medicine, Seoul National University College of Medicine, Seoul, Korea; 3 Department of Nuclear Medicine, Seoul National University Bundang Hospital, Seongnam-si, Gyeonggi-do, Korea; 4 Department of Neuroscience, College of Medicine, Gachon University, Incheon, Korea; 5 Department of Nuclear Medicine, National Cancer Center, Goyang-si, Gyeonggi-do, Korea; 6 Department of Nuclear Medicine, Ewha Womans University School of Medicine, Seoul, Korea; 7 Institute of Radiation Medicine, Medical Research Center, Seoul National University, Seoul, Korea

## Abstract

Quantitative SPECT/CT is potentially useful for more accurate and reliable measurement of glomerular filtration rate (GFR) than conventional planar scintigraphy. However, manual drawing of a volume of interest (VOI) on renal parenchyma in CT images is a labor-intensive and time-consuming task. The aim of this study is to develop a fully automated GFR quantification method based on a deep learning approach to the 3D segmentation of kidney parenchyma in CT. We automatically segmented the kidneys in CT images using the proposed method with remarkably high Dice similarity coefficient relative to the manual segmentation (mean = 0.89). The GFR values derived using manual and automatic segmentation methods were strongly correlated (R2 = 0.96). The absolute difference between the individual GFR values using manual and automatic methods was only 2.90%. Moreover, the two segmentation methods had comparable performance in the urolithiasis patients and kidney donors. Furthermore, both segmentation modalities showed significantly decreased individual GFR in symptomatic kidneys compared with the normal or asymptomatic kidney groups. The proposed approach enables fast and accurate GFR measurement.

## Introduction

Glomerular filtration rate (GFR) is defined as a flow rate of blood plasma that is filtered through glomerulus. It is considered as an indicator of renal function and is routinely used to stratify the severity of acute kidney injury or chronic kidney disease. The GFR is estimated using a substance that is completely filtered through glomerulus, not reabsorbed and not excreted in renal tubules^1^. If that is the case, urinary clearance of the substance is equal to the plasma clearance. Formulas such as Modification of Diet in Renal Disease (MDRD) or Chronic Kidney Disease Epidemiology Collaboration (CKD-EPI) equations are frequently used in the clinic to derive GFR from the serum creatinine level, although creatinine is not an ideal substance for measuring renal clearance^2^.

In nuclear medicine, ^51^Cr-ethylenediaminetetraacetic acid (EDTA) and ^99m^Tc-diethylenetriaminepentaacetic acid (DTPA) are the two most commonly utilized radiopharmaceuticals to evaluate renal function. Blood or urine sampling after injection of the radiotracers is a method to directly measure renal clearance^1,3^. However, these sampling procedures are laborious and time-consuming in comparison with other approaches. Therefore, ^99m^Tc-DTPA planar scintigraphy that measures the radiation counts in each kidney is more commonly used because of its ease of use. The most popular method to calculate the GFR from the radiation counts is Gate’s method that utilizes the counts measured for 1 min from 2 min to 3 min after the injection of ^99m^Tc-DTPA^4,5^.

Kidney single-photon emission computed tomography (SPECT)/computed tomography (CT) with ^99m^Tc-DTPA is a promising method for the measurement of GFR because it is more quantitative and reliable in measuring the renal clearance than planar scintigraphy^6^. The SPECT/CT is acquired during the same period of time as in the renal planar scintigraphy, and the volume of interest (VOI) is drawn on each kidney for the quantification of absolute radioactivity. The measurement of GFR using ^99m^Tc-DTPA SPECT/CT was reproducible and accurate in healthy volunteers and renal tumor patients post partial nephrectomy and useful for disease severity evaluation in urinary stone patients^6,7^. However, the necessity of manual drawing of the VOI on the whole renal parenchyma in CT images is an obstacle that prevents the wide use of this new approach. The labor-intensive and time-consuming manual drawing usually takes about 15 min per scan by nuclear medicine physicians^6,7^.

The aim of this study is to develop an automated GFR quantification method based on deep learning approach to the three-dimensional (3D) segmentation of kidney parenchyma in CT acquired in quantitative kidney SPECT/CT studies. In recent years, deep convolutional neural networks (CNNs) have shown superior performance in many computer vision and biomedical applications, such as image de-noising, image classification and object detection, and organ segmentation^8–18^. There is a related deep-learning-based kidney segmentation study for total kidney volume (functioning parenchyma and non-functioning cysts) quantification in autosomal dominant polycystic kidney disease (ADPKD)^19^. In addition, some studies have shown that deep-learning based 3D segmentation is effective for medical image dataset^20–23^. However, the GFR quantification needs the segmentation of the only functioning kidney parenchyma, which is more sophisticated than total kidney segmentation in ADPKD. In this study, we have trained a deep CNN to learn end-to-end mapping between the 3D CT volume and manually segmented VOI by experts using a dataset including 315 patients. The performance of the CNN was validated using another dataset including 78 patients, and five-fold cross-validation was performed. Finally, the measurement of GFR using the manually drawing and deep-learning-generated VOIs were compared with each other in 63 urolithiasis patients and 25 negative controls (kidney donor) to show the clinical validity of the proposed method.

## Methods

### Dataset

Quantitative ^99m^Tc-DTPA kidney SPECT/CT data of 393 patients (257 men and 136 women, age = 53.55 ± 12.64 years) were retrospectively analyzed for network training and validation (Supplementary Table S1). The retrospective use of the scan data and waiver of consent were approved by the Institutional Review Board of our institute. The SPECT/CT data were acquired using a GE Discovery NM/CT 670 scanner equipped with a low-energy high-resolution collimator^6^. One-minute SPECT data were acquired in a continuous mode 2 min after the intravenous injection of 370 MBq ^99m^Tc-DTPA. The peak energy was set at 140 KeV with 20% window (126–154 KeV), and the scatter energy was set at 120 KeV with 10% window (115–125 KeV). The SPECT images were reconstructed using an iterative ordered subset expectation maximization (OSEM) algorithm (2 iterations and 10 subsets) and corrected for attenuation, scatter, and collimator–detector response. A post-reconstruction low pass filter (Butterworth with frequency of 0.48 and order of 10) was applied and image matrices were 128 × 128 × 128 (voxel size: 3.452 mm^3^). The applied zoom factor during SPECT acquisition was 1.28. The CT acquisition conditions were as follows: tube voltage of 120 KVp, tube current of 60–210 mA with autoMA function at a noise level of 20, detector collimation of 20 mm ( = 16 × 1.25 mm), helical thickness of 2.5 mm, table speed of 37 mm/s, tube rotation time of 0.5 s, and pitch of 0.938:1. The CT images were reconstructed in a 512 × 512 × 161 matrix with voxel sizes of 0.977 × 0.977 × 2.5 mm^3^. The system sensitivity of SPECT for ^99m^Tc was 152.5 cpm/μCi, which had been determined by triple independent sessions of phantom studies^24^.

A nuclear medicine physician manually drew 2D regions of interest (ROIs) on individual renal parenchyma. To cover the whole kidney volume, approximately 80–100 slices were required in normal kidney. To save time and effort, ROIs were drawn in every 2–3 coronal CT slices up to 30 slices using the vendor’s Q Metrix software, excluding unwanted structures like cysts, urinary stones, and tumors. After automatic ROI interpolation between the slices, provided by Q Metrix, a VOI was then generated by integrating these manually drawn and interpolated single-slice ROIs. While the automatic ROI interpolation is useful for reducing the time and labor required for the VOI drawing, the interpolated slices may still include unwanted structures, would then require further manual interventions. It is of note that the quantitative kidney SPECT/CT was performed without iodine-contrast enhancement; however, iodine-contrast remained in the renal pelvis of the SPECT/CT in 22.6% ( = 89/393) because contrast-enhanced CT was performed 1–2 hours prior to the SPECT/CT for the post-operative evaluation of renal tumor **(**Supplementary Table S1**)**.

**Figure 1:**
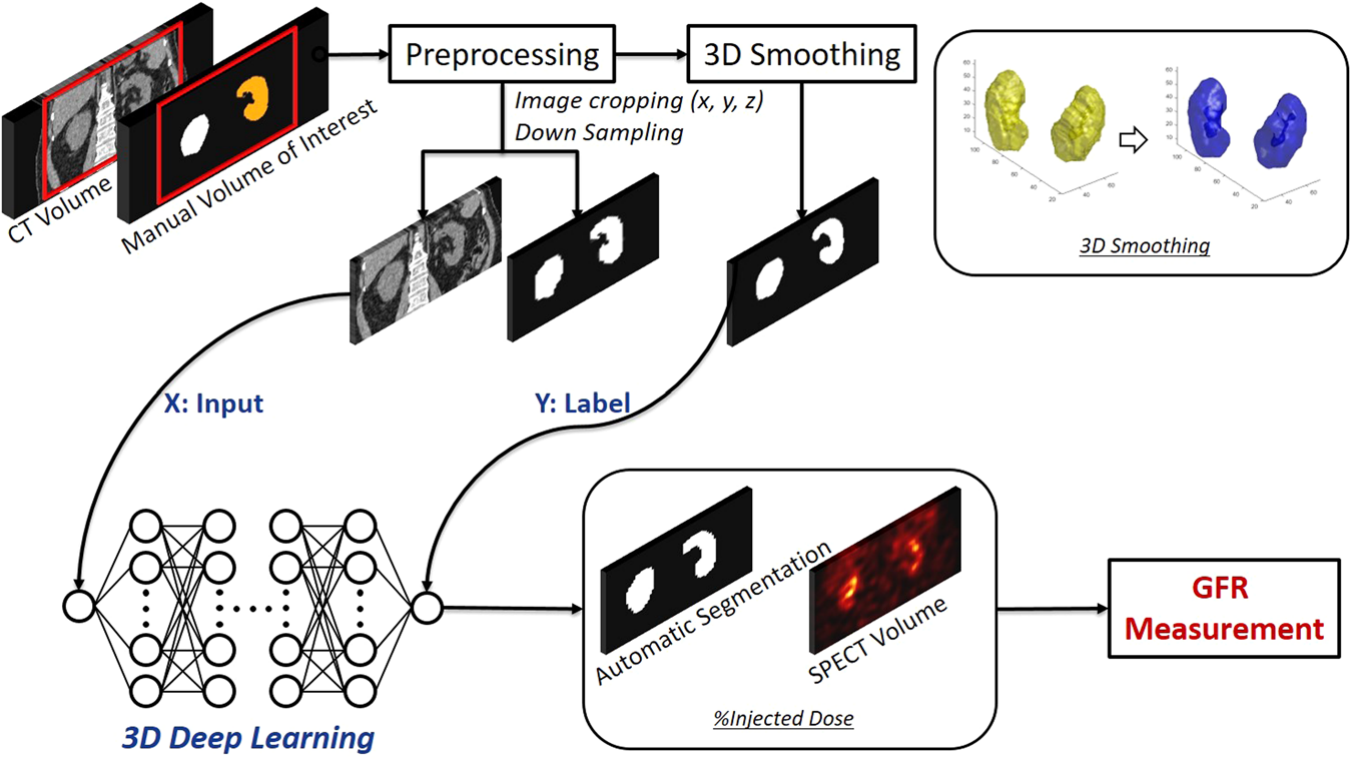
Schematic diagrams of the deep-learning-based renal parenchyma segmentation for the measurement of glomerular filtration rate (GFR) using quantitative single-photon emission computed tomography (SPECT)/computed tomography (CT).

For CNN training and data analysis, CT and SPECT images and manual VOIs were resampled to have the same matrix and voxel sizes (256 × 256 × 232 and 1.726 mm^3^). To reduce the memory consumption in CNN training, the images were then cropped into 192 × 128 × 96 matrices, which are large enough to include both kidneys. In addition, we applied 3D volume smoothing and morphological operations to the manual VOIs to reduce the discontinuity in 3D space caused by the 2D ROI drawing. These preprocessed images and VOIs were finally used for CNN training and testing and GFR estimation as shown in Fig. 1.

### Neural network architecture

The CNN that we used is a modified 3D U-net that consists of the contraction and expansion paths^25^. The 3D U-net learns an end-to-end mapping between CT and manually drawn renal parenchyma VOIs as shown in Supplementary Figure S1. Each path is exploited by the five sequential layers. The contraction path, which captures the context, consist of a leaky rectified linear unit (leaky ReLU) as an activation function (a pre-activation residual block^26^), each followed by 3 × 3 × 3 convolution and 2 × 2 × 2 strided convolution for down-sampling. In addition, the element-wise sum array is used between the output of 2 × 2 × 2 strided convolution and 3 × 3 × 3 convolution to forward feature maps from one stage of the network to other^20^. The expansion path, which enables precise localization, consists of the leaky ReLU, which allows a small gradient when the unit is not active, 3 × 3 × 3 convolution, 1 × 1 × 1 convolution, and 2 × 2 × 2 de-convolution for up-sampling. The element-wise sum array layer is also used right before the Sigmoid activation function to sum 3 × 3 × 3 convolution results of the previous three layers^20^. We used a 3D spatial drop-out technique (drop-out rate of 0.3), as these have shown better performance when adjacent voxels within feature maps are strongly correlated compared with batch normalization.

We also employed symmetric skip connections (copy and concatenation), as shown in Supplementary Figure S1, to insert the local details captured in the feature maps of the contraction path into the feature maps of the expansion path^27^. We implemented the networks using the TensorFlow^28^ and Keras framework (https://github.com/fchollet/keras).

### Network training

The network was trained using randomly selected dataset of 315 of 393 patients and validated using data from the remaining 78 patients. As mentioned previously, the resampled and cropped CT images and manual VOIs were used for network training. All the input and output datasets were in 3D volume format.

The Dice similarity coefficient, which is an overlap metric frequently used for assessing the quality of segmentation maps, is used as the loss function^11,21,29^. Each layer was updated using error back-propagation with adaptive moment estimation optimizer (ADAM), which is a stochastic optimization technique^30^. The exponential decay rates for the moment estimates *β*_1_ and *β*_2_ are 0.9 and 0.999 respectively, with epsilon of zero. The learning rate for determining to what extent the newly acquired information overrides the old information was initially 0.0005 and reduced by half after 10 epochs if the loss function is not improved. The number of epochs was 80 and each epoch includes 272 iterations. The training time was approximately 60 min/epoch when using i7-7700K CPU (3.40 GHz) and one GTX 1080 TI GPU.

### GFR estimation

We calculated %ID by applying the manual and automatic VOIs to the quantitative SPECT images. Then, individual GFRs were calculated using the following equation^6^:

$${\rm{GFR}}\,({\rm{ml}}/{\rm{\min }})=( \% \mathrm{ID}\times 9.1462)+23.0653$$

The sum of bilateral kidney GFRs was normalized to body surface area (BSA) using the following equation to calculate the total GFR of the bilateral kidneys. The Dubois equation for the BSA in m^2^ was 0.007184 × (weight in kg)^0.425^ × (height in cm)^0.725^.

$${\rm{Total}}\,{\rm{GFR}}\,({\rm{ml}}/{\rm{\min }}/1.73\,{{\rm{m}}}^{2})={\rm{GFR}}\,({\rm{ml}}/{\rm{\min }})\times (1.73/{\rm{BSA}}\,{{\rm{m}}}^{2})\,$$

### Further validation on urolithiasis patients

To evaluate the performance of the network in the clinical setting, we adopted patients with urinary stones and kidney donors as negative controls. Consecutive ^99m^Tc-DTPA kidney SPECT/CT studies of urolithiasis patients or kidney donors performed from March 2015 to January 2016 were analyzed retrospectively. Among 69 urinary stone patients scanned during that period, 4 with underlying chronic kidney disease and 2 without available raw data were excluded. Among 26 kidney donors, one subject without raw data remaining was also excluded. Finally, 126 kidneys from 63 urinary stone patients and 50 kidneys from 25 kidney donors were investigated. Gender proportion was not significantly different between the normal (male:female = 15:10) and stone group (male:female = 30:33) (Chi-square test, *p* = 0.086). However, age was significantly higher in urinary stone subjects (56.87 ± 12.60 years old) than in normal subjects (45.64 ± 13.91 years old) (independent samples *t*-test, *p = *0.0004).

Each kidney was classified into three groups: 50 normal kidneys (from kidney donor patients), 48 symptomatic kidneys with either ureter stone of any size or large renal stone (longest diameter > 10 mm), and 78 asymptomatic kidneys with either small renal stone (longest diameter ≤ 10 mm) or contralateral kidney of unilateral urolithiasis patients.

Individual and total GFR values obtained from manual segmentation or automatic segmentation were compared in each group. For manual segmentation, the average of two independent measurements of GFR by four medical experts was used to represent the manual GFR. The experts were blind to each other regarding the manual segmentation results. For automatic segmentation, only a single measurement of GFR by the deep learning algorithm was employed.

### Data analysis

For the quantitative evaluation of network performance, the Dice similarity coefficient between manual drawing and deep learning output was calculated. In addition, we assessed the correlation and mean absolute percentage error between the measurements of GFR using these different segmentation methods. To confirm the consistency of performance, we also performed five-fold cross-validation.

Statistical analyses in urolithiasis patients were performed with dedicated software (Medcalc, version 14.8.1, bvba/GraphPad Prism, version 5.01). First, normality of the data was evaluated using the D’Agnostino–Pearson test and parametric or non-parametric tests were implemented according to the result. For the parametric test, independent samples *t*-test, paired samples *t*-test, or one-way analysis of variance (ANOVA) was performed. For the non-parametric test, Mann–Whitney test, Wilcoxon test, or Kruskal–Wallis test was done. Chi-square tests were performed for analyses of categorical data. A multiple comparison correction for *t*-test was implemented with Bonferroni correction. Results with *P*-values less than 0.05 were considered significant.

## Results

### Segmentation

We could automatically segment the kidneys in CT images using the proposed method with high Dice similarity coefficient relative to the manual segmentation (mean ± SD = 0.89 ± 0.03 in main experiment) (Table 1). In addition, the proposed deep learning approach provided 3D kidney parenchyma VOIs with no discontinuity between slices because the CNN was trained to produce smooth 3D VOIs. The time requirement of auto-segmentation was only a few seconds per patient, whereas the manual segmentation takes about 15 min per scan. We also performed an ablation study to optimize the network structure. The results from the ablation study is summarized in Supplementary Table S2. Due to memory limitation, we could not use more than one batch without additional down-sampling of the image dataset. We observed that using the drop-out without down-sampling (batch size of one) showed better performance than batch normalization with down-sampling (batch size of two). Using the residual block and the element-wise sum array increased the Dice similarity coefficient. In addition, the dice coefficient was slightly improved by applying the drop-out for the proposed network.

**Table 1:** The results of cross-validations (total kidney).

Method	Unit	Dataset				
		Main Experiment	Cross-validation 1	Cross-validation 2	Cross-validation 3	Cross-validation 4
DSC	(mean ± SD)	0.89 ± 0.03	0.88 ± 0.04	0.88 ± 0.04	0.89 ± 0.03	0.89 ± 0.03
	[range]	0.80–0.93	0.65–0.93	0.74–0.94	0.77–0.93	0.79–0.94
Mean-M	ml/min (mean ± SD)	49.87 ± 10.08	49.28 ± 10.21	47.83 ± 9.58	50.27 ± 10.72	49.68 ± 10.06
Mean-A	ml/min (mean ± SD)	49.41 ± 9.81	48.89 ± 9.88	47.70 ± 9.07	49.95 ± 10.26	49.06 ± 9.68
Correlation	*R* ^2^	0.96	0.96	0.95	0.96	0.96
MAPE	% (mean ± SD)	2.90 ± 2.80	2.88 ± 2.75	2.99 ± 3.25	3.00 ± 2.93	2.67 ± 2.70

DSC, Dice similarity coefficient; M, manual segmentation; A, automatic segmentation; MAPE, mean absolute percentage error.

**Figure 2:**
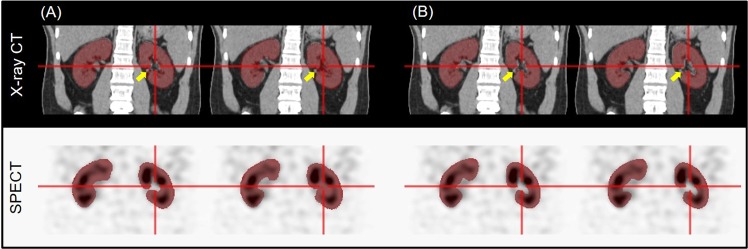
Single-photon emission computed tomography (SPECT)/computed tomography (CT) images and renal parenchyma volumes of interest (VOIs) with incorrect region of interest (ROI) interpolation result (next slice as the second and fourth column) provided from the vendor’s software. (**A**) Manually segmented VOI. (**B**) Deep-learning-generated automatic VOI.

**Figure 3:**
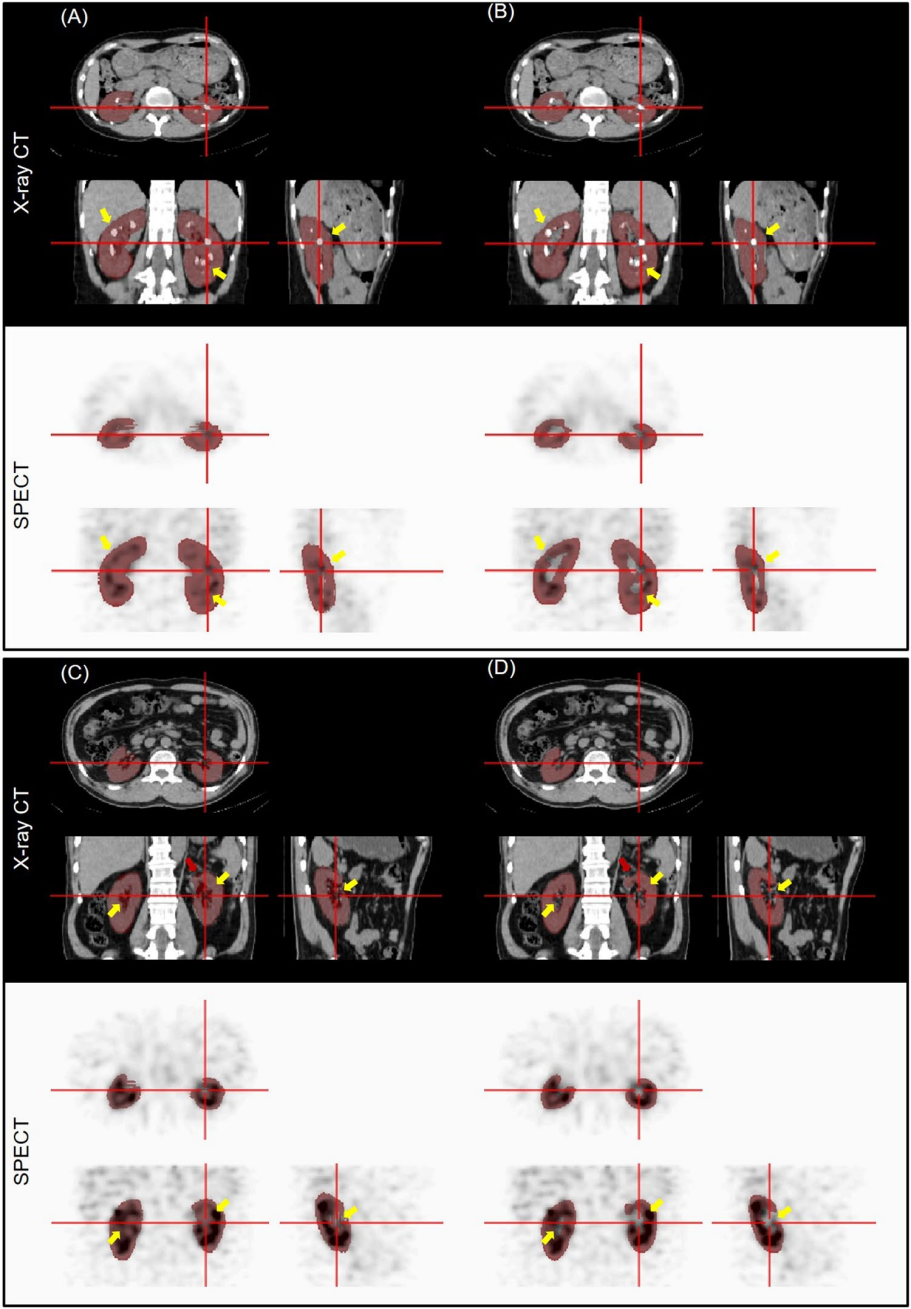
Single-photon emission computed tomography (SPECT)/computed tomography (CT) images (multiple renal stones for (**A**,**B**) and partial nephrectomy for (**C**,**D**)) and renal parenchyma volumes of interest (VOIs) for a representative test dataset (**A**,**C**) Manually segmented VOI. (**B**,**D**) Deep-learning-generated automatic VOI.

Figures 2, and 3 show some cases in which the CNN outperformed the manual segmentation that was supported by the automatic inter-slice ROI interpolation function provided by the vendor’s software. Note that the errors are mainly associated to the time-consuming nature of the manual segmentation in which every frame was not segmented as a compromise.

In Fig. 2, the pelvis of the left kidney is wrongly included in the manual VOI (Fig. 2A) although the CNN did not yield such error (Fig. 2B). Figure 3 shows another case in which the CNN well excludes the multiple renal stones (yellow arrow in Fig. 3B), but the manual VOI failed to exclude multiple stones (Fig. 3A). In Fig. 3C,D, the CNN well delineates the partial nephrectomy margin in the left kidney (red arrow). In addition, the accuracy of segmentation was better in the CNN outcome (yellow arrow).

### GFR estimation

**Figure 4:**
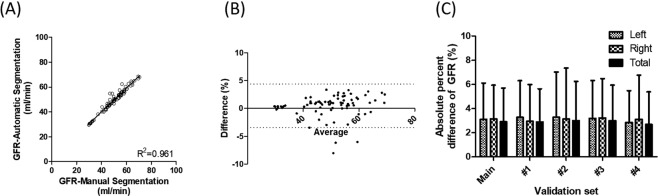
Scattered (**A**) and Bland–Altman (**B**) plots between measurement of total glomerular filtration rate (GFR) using manual and deep-learning-generated volumes of interest (VOIs), and absolute percentage difference (**C**) between measurement of GFR using manual and deep-learning-generated VOIs: results of five-fold cross-validation.

The GFR values derived using manual and automatic segmentation methods were strongly correlated (*R*^2^ = 0.96 in main experiment) for total kidneys (Table 1). Scattered and Bland–Altman plots between the measurement of GFR in total kidneys using manual and deep-learning-generated VOIs are shown in Fig. 4A,B. The result of each right and left kidney is shown in Supplementary Figure S2.

Figure 4C shows the percentage difference (mean absolute percentage error) between the measurements of GFR obtained using manual and deep-learning-generated VOIs in all five-fold cross-validations. The absolute difference between the GFR values using manual (49.87 ± 10.08 ml/min) and automatic (49.41 ± 9.81 ml/min) methods was only 2.90 ± 2.80% (left kidney: 3.12 ± 2.99%; right kidney: 3.13 ± 2.80%) in the main experiment. The percentage differences obtained in the other cross-validations were 2.88 ± 2.75%, 2.99 ± 3.26%, 3.00 ± 2.94%, and 2.68 ± 2.70%, respectively. The results of cross-validations are summarized in Table 1. The correlation coefficient *R*^2^ for the five sets ranged from 0.95 to 0.96.

### Validation on urolithiasis patients

The CNN segmentation-based GFR (GFR_CNN_) was applied for further clinical validation for urolithiasis patients. The manual-segmentation-based GFR by four human experts (GFR_manual_) served as a reference. Individual kidney GFR and total GFR (sum of bilateral GFR with body surface area normalization) were investigated.

Supplementary Figure S3A and Table 2 show that GFR_CNN_ and GFR_manual_ were equivalent in terms of total GFR evaluation of urolithiasis and controls. Total GFR_CNN_ in kidney donors (119.25 ± 18.35 ml/min/1.73 m^2^) was not significantly different from GFR_manual_ (120.39 ± 19.26 ml/min/1.73 m^2^; *P* = 0.4432, Wilcoxon test). Total GFR_CNN_ in urinary stone patients (115.02 ± 17.71 ml/min/1.73 m^2^) was also not significantly different from GFR_manual_ (115.65 ± 16.91 ml/min/1.73 m^2^; *P* = 0.2387, paired *t*-test). Meanwhile, total GFR in the normal and stone groups showed no significant difference in both manual (*P* = 0.2582, independent samples *t*-test) and CNN-based segmentations (*P* = 0.5693, Mann–Whitney test).

**Table 2:** Total glomerular filtration rate (GFR) (ml/min/1.73 m^2^) by manual and convolutional neural network (CNN)-based segmentations in normal and urolithiasis patients (mean ± SD).

	Normal (*n* = 25)	Stone (*n* = 63)	*P*-value
Manual	120.39 ± 19.26	115.65 ± 16.91	*NS*
CNN	119.25 ± 18.35	115.02 ± 17.71	*NS*
*P*-value	*NS*	*NS*	

NS, non-significant.

When it comes to the individual kidney GFR, GFR_CNN_ and GFR_manual_ were comparable with each other without significant difference. Supplementary Figure S3B and Supplementary Table S3 show that individual GFR_CNN_ in normal kidneys (60.43 ± 7.66 ml/min) was not significantly different from GFR_manual_ (61.01 ± 8.10 ml/min; *P* = 0.1725, paired *t*-test). In addition, individual GFR was not significantly different in asymptomatic kidneys (GFR_CNN_: 59.23 ± 9.25 ml/min versus GFR_manual_: 59.72 ± 9.46 ml/min; *P* = 0.0361, paired *t*-test) and in symptomatic kidneys (GFR_CNN_: 51.76 ± 13.69 ml/min versus GFR_manual_: 51.84 ± 12.73 ml/min).

Individual GFR in normal, asymptomatic, and symptomatic kidneys were significantly different in both manual (*P < *0.001, ANOVA) and CNN-based segmentation methods (*P* < 0.001, ANOVA). *Post-hoc* analyses revealed that in both manual and CNN segmentations, symptomatic kidneys had significantly lower GFR compared with normal or asymptomatic kidneys (*P* < 0.05).

**Figure 5:**
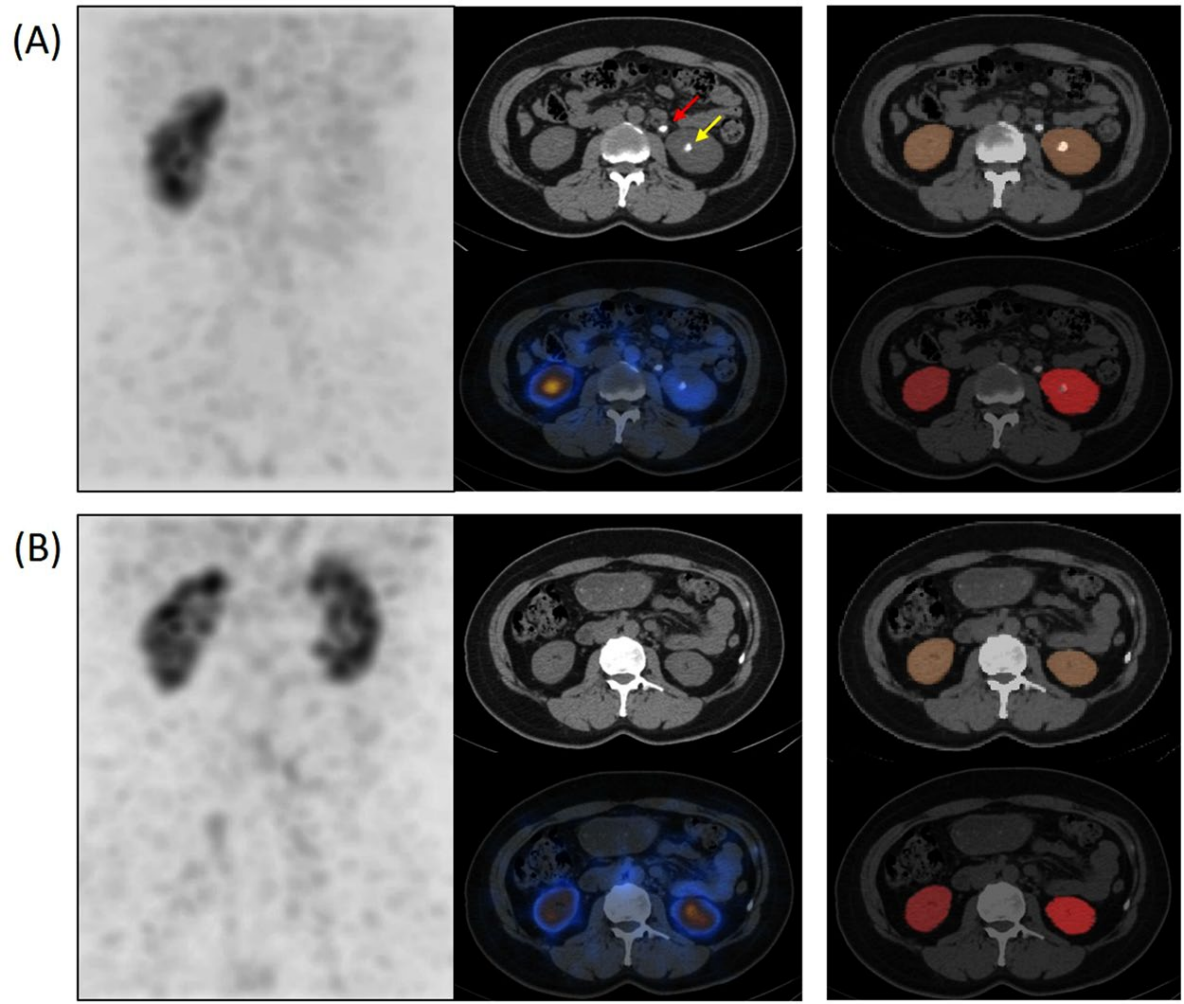
Single-photon emission computed tomography (SPECT)/computed tomography (CT) images of a patient (**A**) before (red arrow indicates a ureter stone and yellow arrow indicates a renal stone) and (**B**) 4 months after removal of left ureter and renal stones. A projection image is presented in the first column, axial images of CT (top) and SPECT/CT fusion (bottom) are in the second, and segmentation results (automatic segmentation in the top and manual segmentation in the bottom) are shown in the third column.

Finally, manual and automatic segmentation methods showed comparable performance in an evaluation of treatment response. Figure 5 and Supplementary Table S4 present a typical case of a urinary stone patient before and after removal of ureter and renal stones in a left kidney. In serial projection images, ^99m^Tc-DTPA uptake in left renal parenchyma is normalized after the procedure. Both manual and automatic segmentation methods showed marked improvement of %ID and individual GFR in the left kidney after the removal of the stones.

## Discussion

In this study, we showed that the deep learning approach is highly accurate in renal parenchyma segmentation in CT images acquired in kidney SPECT/CT studies and is useful for automated measurement of GFR. The CNN outcomes yielded remarkably high Dice coefficient (0.89) with manual segmentation, leading to the strong correlations in %ID and GFR between the manual and automatic methods.

Automatically drawing VOIs only on renal parenchyma but excluding cysts and tumors is a challenging task because their CT intensities are very similar in non-contrast-enhanced CT images obtained in SPECT/CT studies. Although the proposed method performed the segmentation correctly in most cases as shown in Supplementary Figure S4, there were several cases in which the segmentation was not accurate. Supplementary Figure S5 is such a case in which a renal mass (yellow arrows) was incorrectly included although renal pelvis was well excluded (red arrows). Because this patient (male, 164 cm, 58 kg) was relatively smaller than others, insufficient data for training deep CNN to properly handle such unusual cases would be the cause of inaccurate segmentation. In spite of such inaccuracy, the GFR error in this patient was only 2.48% because the radioactivity in the tumor was very low.

Because we trained the CNN to draw VOIs on the renal parenchyma of both kidneys, there was error in the patient with only a single kidney. In Supplementary Figure S6, the CNN drew a long narrow VOI on the liver parenchyma (yellow arrow) of a patient who does not have a right kidney. The CNN experienced only three single-kidney cases during the training among the training set with 272 patients. Additional datasets of with single-kidney patients will be necessary to overcome this limitation. In the cases shown in Figs 2 and 3, there were some segmentation errors in manual VOIs. By carefully inspecting the cause of these errors, we could reveal that the error in the manual VOI originated from the discontinuity in the perpendicular direction to the ROIs and subsequent inter-slice ROI interpolation. In two sequential slices shown in Fig. 2A, the ROI on the left was manually drawn and that on the right was interpolated.

In the further validation of the proposed method for urolithiasis, automatic segmentation was comparable with manual segmentation in measuring GFR. There was no difference between manually driven GFR and CNN-driven GFR in all groups of patients (total GFR) and kidneys (individual GFR). In addition, in both automatic and manual segmentation methods, individual kidneys with symptomatic urinary stone had lower GFR compared with normal or kidneys with asymptomatic stones. We could presume that both segmentation methods work well to represent the functional deterioration by obstructing urinary stones and the subsequent improvement after stone removal procedures^31,32^. Further clinical validation of deep-learning-based segmentation is required to expand its use in more complicated cases such as multi-cystic dysplastic kidneys where manual segmentation is more laborious.

To the best of our knowledge, this is the first deep learning study on the kidney parenchyma segmentation in CT and its application to the SPECT activity quantification. The proposed deep learning approach to the 3D segmentation of kidney parenchyma in CT enables fast and accurate measurement of GFR. The combination of CT-based automatic segmentation by the deep learning approach and novel quantitative SPECT technology may pave the way for precision nuclear medicine regarding measurement of GFR.

## Supplementary information

Supplementary Figure S1 - S6, Supplementary Table S1 - S4
