# Supplementary material for: Measurement of Glomerular Filtration Rate using Quantitative SPECT/CT and Deep-learning-based Kidney Segmentation
Source: Sci Rep. 2019 Mar 12;9:4223. doi: 10.1038/s41598-019-40710-7 (PMC6414660; doi:10.1038/s41598-019-40710-7)
Supplement: Supplementary file 1 — Supplementary Figure S1 - S6, Supplementary Table S1 - S4 [file 41598_2019_40710_MOESM1_ESM.docx]

**Measurement of Glomerular Filtration Rate using Quantitative SPECT/CT and Deep-learning-based Kidney Segmentation**

**Junyoung Park^1,2+,^ , Sungwoo Bae^2,3+,^ , Seongho Seo^4^, Sohyun Park^5^, Ji-In Bang^6^, Jeong Hee Han^3^, Won Woo Lee^2,3,7*^, and Jae Sung Lee^1,2,7*^**

^1^Department of Biomedical Sciences, Seoul National University College of Medicine, Seoul, Korea

^2^Department of Nuclear Medicine, Seoul National University College of Medicine, Seoul, Korea

^3^Department of Nuclear Medicine, Seoul National University Bundang Hospital, Seongnam-si, Gyeonggi-do, Korea

^4^Department of Neuroscience, College of Medicine, Gachon University, Incheon, Korea

^5^Department of Nuclear Medicine, National Cancer Center, Goyang-si, Gyeonggi-do, Korea

^6^Department of Nuclear Medicine, Ewha Womans University School of Medicine, Seoul, Korea

^7^Institute of Radiation Medicine, Medical Research Center, Seoul National University, Seoul, Korea

^*^Corresponding author. E-mail: [jaes@snu.ac.kr](mailto:jaes@snu.ac.kr), [wwlee@snu.ac.k](mailto:wwlee@snu.ac.kr)r

^+^Contributed equally for this work.

**
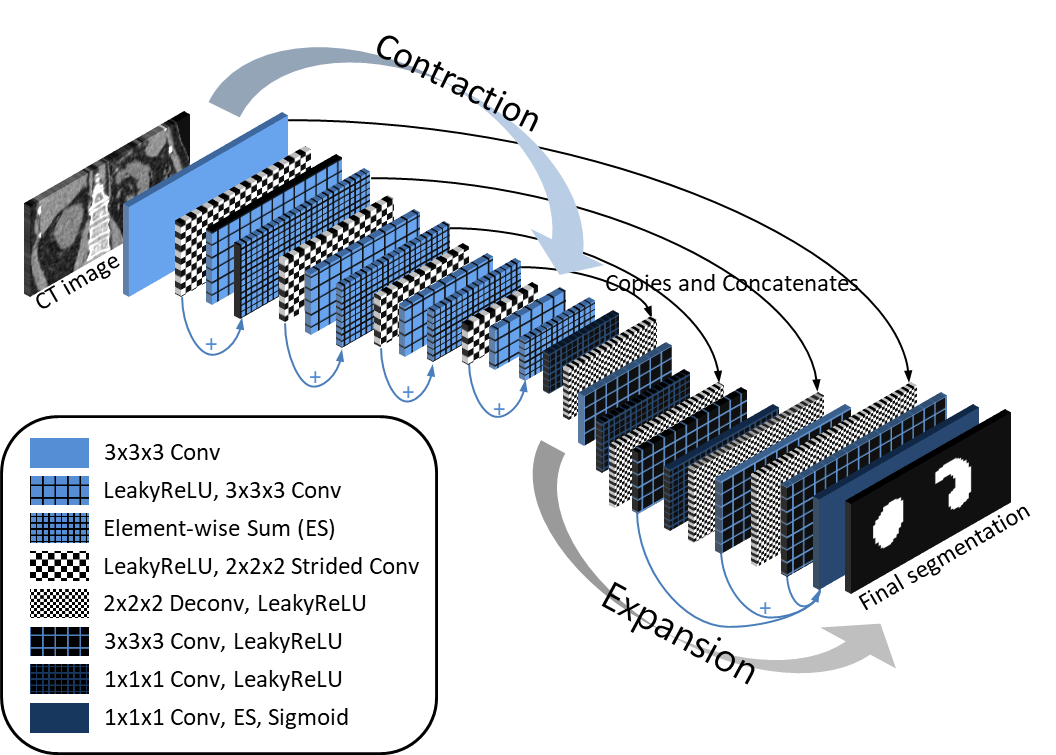
**

**Supplementary Figure S1.** Deep neural network architecture. The network learns an end-to-end mapping between computed tomography (CT) and renal parenchyma segmented volumes. The network consists of the contraction and expansion paths.

**
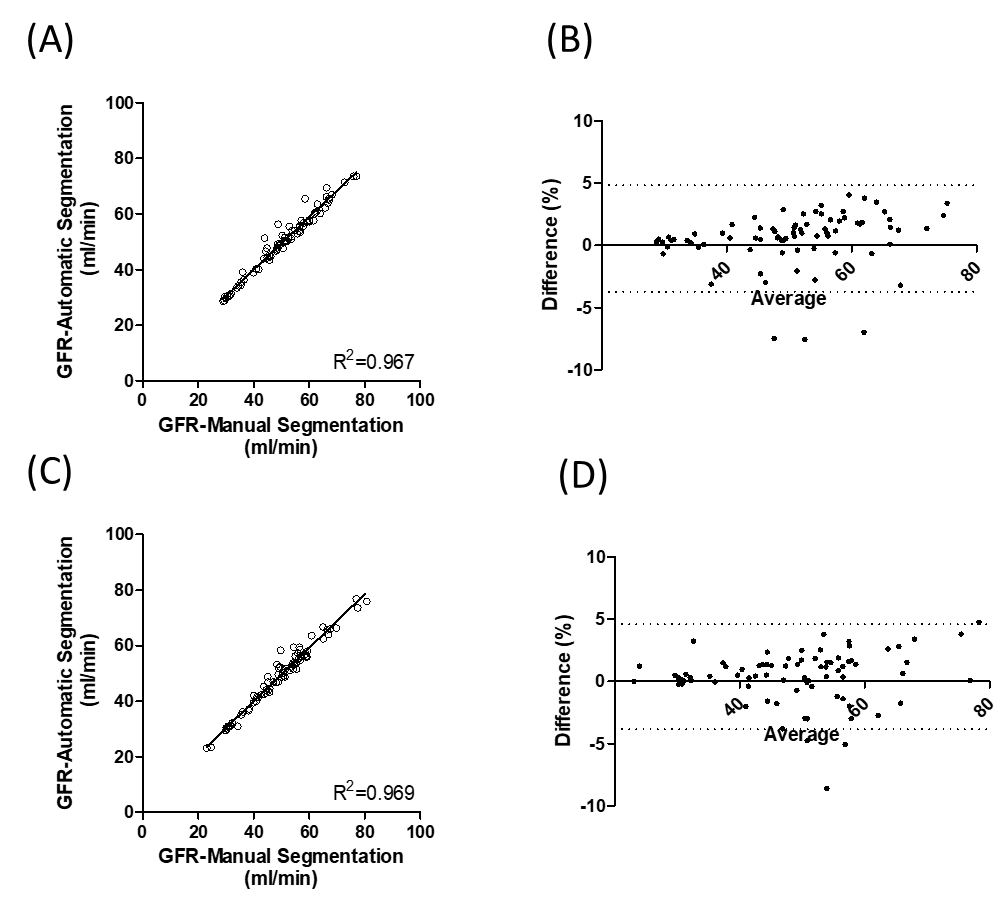
**

**Supplementary Figure S2.** Scattered (A) and Bland–Altman (B) plots between measurement of left glomerular filtration rate (GFR) using manual and deep-learning-generated volumes of interest (VOIs) and scattered (C) and Bland–Altman (D) plots between measurement of right GFR.

**
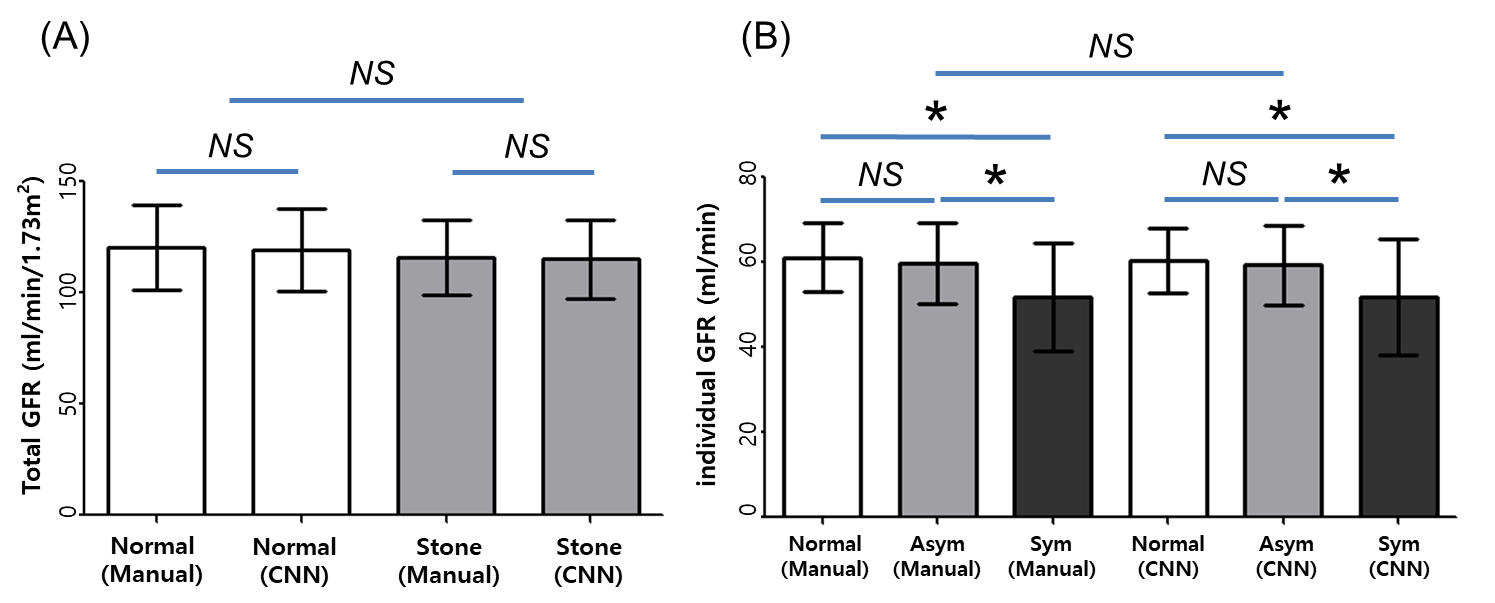
**

**Supplementary Figure S3.** Total and Individual glomerular filtration rate (GFR) comparison. (A) Total GFR comparison of manual and convolutional neural network (CNN)-based automatic segmentation methods in kidney donor (normal) and urinary stone patients (stone). (B) Individual GFR comparison of normal, asymptomatic (Asym), or symptomatic (Sym) kidneys in manual and automatic segmentation methods. *P < 0.001.

**
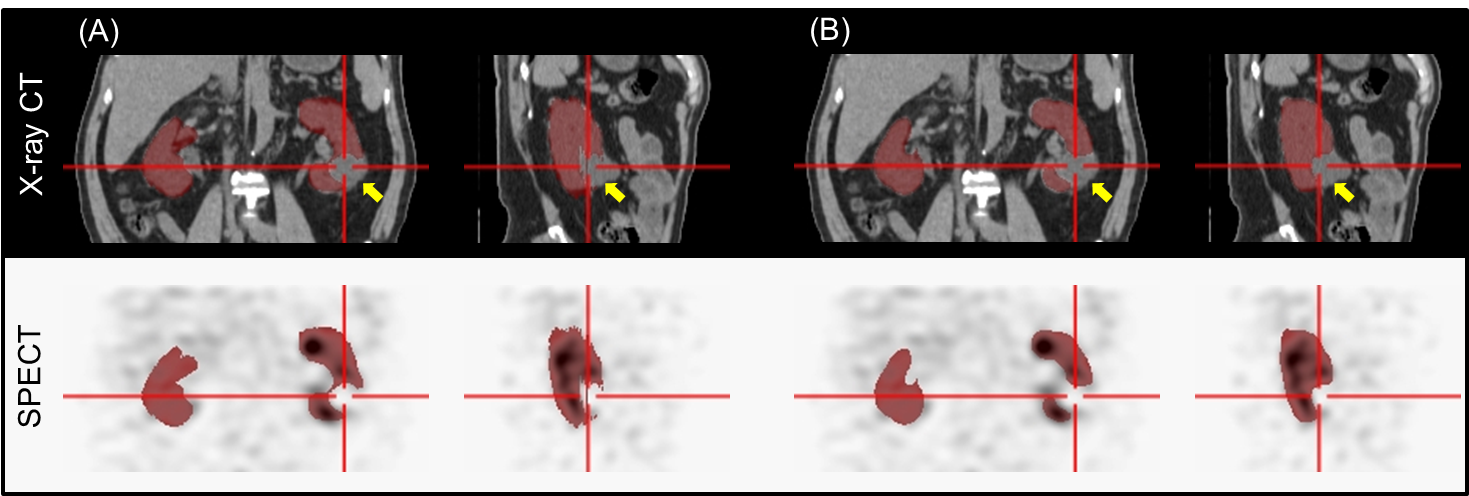
**

**Supplementary Figure S4.** Single-photon emission computed tomography (SPECT)/computed tomography (CT) images (cyst included) and renal parenchyma volumes of interest (VOIs) for a representative test dataset. (A) Manually segmented VOI. (B) Deep-learning-generated automatic VOI.

**
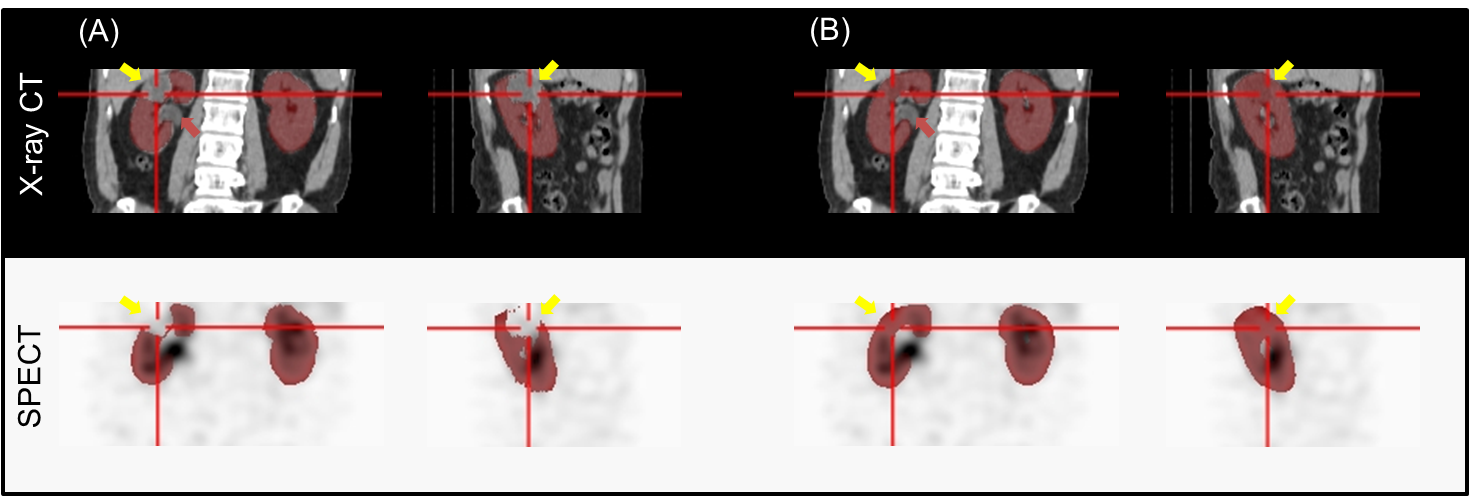
**

**Supplementary Figure S5.** Single-photon emission computed tomography (SPECT)/computed tomography (CT) images (renal mass and renal pelvis included) and renal parenchyma VOIs for a representative test dataset. (A) Manually segmented VOI. (B) Deep-learning-generated automatic VOI.

**
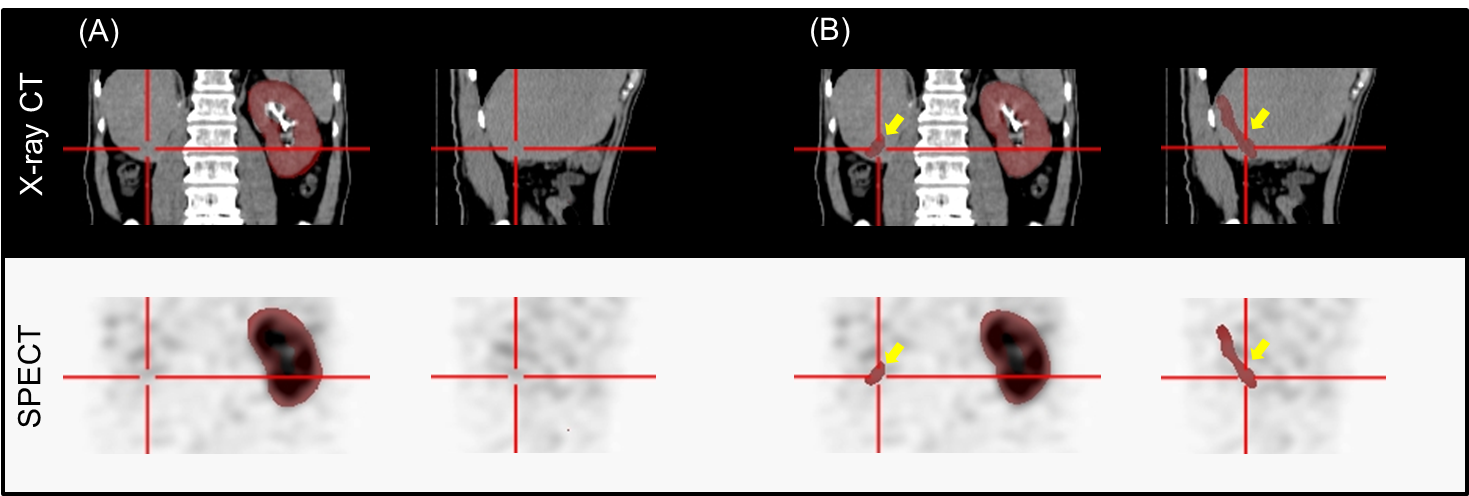
**

**Supplementary Figure S6.** Single-photon emission computed tomography (SPECT)/computed tomography (CT) images (single kidney) and renal parenchyma volumes of interest (VOIs) for a representative test dataset. (A) Manually segmented VOI. (B) Deep-learning-generated automatic VOI.

| Patient Characteristics | Data | |
| --- | --- | --- |
| Gender (male:female) | 257:136 | |
| Age (years) | 53.55 ± 12.64 | |
| Height (cm) | 166.04 ± 9.23 | |
| Weight (kg) | 70.17 ± 12.86 | |
| Body surface area* (m^2^) | 1.78 ± 0.19 | |
| Serum Creatinine (mg/dl) | 0.89 ± 0.26 | |
| eGFR† (ml/min/1.73 m^2^) | 90.63 ± 18.18 | |
| Disease | Normal  (kidney donor) | 18 |
|  | Renal tumor | 83 |
|  | Urinary stone | 112 |
|  | Post partial nephrectomy‡ | 174 |
|  | Post total nephrectomy§ | 5 |
|  | Other | 1 |
| Contrast-enhanced CT (yes:no) | 89:304 | |

* The equation for body surface area is the Dubois formula:

Body surface area (m^2^) = 0.007184 × (weight in kg)^0.425^ × (height in cm)^0.725^

† Estimated glomerular filtration rate (eGFR) is derived from the Chronic Kidney Disease Epidemiology Collaboration (CKD-EPI) equation.

‡ Cause of partial nephrectomy: renal tumor (173) or simple cyst (1).

§ Cause of total nephrectomy: renal tumor (5)

Data=mean ± standard deviation. CT, computed tomography.

**Supplementary Table S1.** Demographics of patients for network training and validation (*n* = 393)

| Model | Version | Average Dice Score |
| --- | --- | --- |
| 3D U-Net | + Down-sampling (x2) + Batch normalization (batch size = 2) | 0.848 |
|  | + Drop-out (P_drop_* = 0.3) | 0.862 |
|  | + Residual block, Element-wise sum array | 0.885 |
|  | + Residual block, Element-wise sum array + Drop-out (P_drop_ = 0.3) | **0.890** |

* P_drop_: Drop-out rate

**Supplementary Table S2.** Ablation study between original 3D U-Net and proposed network. Note that, batch normalization cannot be used without additional down-sampling of the image dataset because of memory limitation.

|  | Normal  (*n* = 50) | Asymptomatic (*n* = 78) | Symptomatic (*n* = 48) | *P*-value |
| --- | --- | --- | --- | --- |
|  |  |  |  |  |
| Manual | 61.01 ± 8.10 | 59.72 ± 9.46 | 51.84 ± 12.73 | <0.001 |
|  |  |  |  |  |
| CNN | 60.43 ± 7.66 | 59.23 ± 9.25 | 51.76 ± 13.69 | <0.001 |
|  |  |  |  |  |
| *P*-value | *NS* | *NS* | *NS* |  |

**P*-value less than 0.05/3 is considered significant in a comparison of manual versus CNN data (Bonferroni correction).

NS, non-significant.

**Supplementary Table S3.** Individual glomerular filtration rate (GFR; ml/min) by manual and convolutional neural network (CNN)-based segmentations in normal, symptomatic, and asymptomatic urinary stone kidneys (mean ± SD)

|  | Manual | | | | CNN | | | |
| --- | --- | --- | --- | --- | --- | --- | --- | --- |
|  | %ID | | GFR (g/ml) | | %ID | | GFR (g/ml) | |
|  | Right | Left | Right | Left | Right | Left | Right | Left |
| Before surgery | 4.13 | 1.54 | 60.84 | 37.15 | 4.22 | 1.64 | 61.64 | 38.05 |
| After surgery | 3.21 | 2.88 | 52.42 | 49.41 | 3.45 | 3.12 | 54.61 | 51.62 |

**Supplementary Table S4.** Change of individual kidney GFR before and after stone removal procedure in a urolithiasis patient.
